# Comprehensive Proteomic Profiling of Urinary Exosomes and Identification of Potential Non-invasive Early Biomarkers of Alzheimer’s Disease in 5XFAD Mouse Model

**DOI:** 10.3389/fgene.2020.565479

**Published:** 2020-11-05

**Authors:** Zhiqi Song, Yanfeng Xu, Ling Zhang, Li Zhou, Yu Zhang, Yunlin Han, Xianglei Li, Pin Yu, Yajin Qu, Wenjie Zhao, Chuan Qin

**Affiliations:** NHC Key Laboratory of Human Disease Comparative Medicine, Beijing Key Laboratory for Animal Models of Emerging and Remerging Infectious Diseases, Institute of Laboratory Animal Science, Chinese Academy of Medical Sciences and Comparative Medicine Center, Peking Union Medical College, Beijing, China

**Keywords:** urinary exosome proteome, 5XFAD mouse model, Alzheimer’s disease, biomarkers, early diagnosis

## Abstract

**Background:**

Alzheimer’s disease (AD) is an incurable neurodegenerative disease characterized by irreversible progressive cognitive deficits. Identification of candidate biomarkers, before amyloid-β-plaque deposition occurs, is therefore of great importance for early intervention of AD.

**Objective:**

To investigate the potential non-invasive early biomarkers of AD in 5XFAD mouse model, we investigate the proteome of urinary exosomes present in 1-month-old (before amyloid-β accumulation) 5XFAD mouse models and their littermate controls. Another two groups of 2 and 6 months-old urinary samples were collected for monitoring the dynamic change of target proteins during AD progression.

**Methods:**

Proteomic, bioinformatics analysis, multiple reaction monitoring (MRM), western blotting (WB) or ELISA were performed for analyzing these urinary exosomes.

**Results:**

A total of 316 proteins including 44 brain cell markers were identified using liquid chromatography tandem mass spectrometry. Importantly, 18 proteins were unique to the 5XFAD group. Eighty-eight proteins including 11 brain cell markers were differentially expressed. Twenty-two proteins were selected to be verified by WB. Furthermore, based on an independent set of 12 urinary exosomes samples, five in these proteins were further confirmed significant difference. Notably, Annexin 2 and Clusterin displayed significant decreased in AD model during the course detected by ELISA. AOAH, Clusterin, and Ly86 are also brain cell markers that were first reported differential expression in urinary exosomes of AD model.

**Conclusion:**

Our data demonstrated that some urinary exosome proteins, especially Annexin 2 and Clusterin, as nanometer-sized particles, enable detection of differences before amyloid-β-plaque deposition in 5XFAD mouse model, which may present an ideal non-invasive source of biomarkers for prevention of AD.

## Introduction

Alzheimer’s disease is a chronic age-associated neurodegenerative disease related to irreversible cognitive impairment and progressive dementia (Dos et al., 2018). As the pathological course causing AD starts decades before clinical symptoms appear, identifying clues in the early stages of AD, especially before amyloid-β (Aβ) plaque deposition, is urgent for AD research (Dubois et al., 2015). Urine is an ideal resource to discover new biomarkers not only for kidney-related disease, but also for non-kidney-related diseases (Dubois et al., 2015), including AD diagnosis (Zhang et al., 2018). Additionally, urine can be collected non-invasively in large amounts. However, the protein concentration in normal urine is very low compared with other body fluids, and normal protein excretion is < 150 mg/day or 100 mg/L (Adachi et al., 2006). The presence of highly abundant proteins (e.g., albumin) may mask the identification of under-represented proteins that have potential pathophysiological significance (Adachi et al., 2006). Exosomes are small extracellular nano-sized vesicles between 40 and 160 nm in diameter (Kalluri and LeBleu, 2020) that were first described in the 1980s (Johnstone et al., 1987). They can be isolated from a variety of body fluids, making them amenable to proteomic and transcriptomic analyses; this supports the hypothesis that their analysis may provide valuable information for disease diagnosis and monitoring (Mitchell et al., 2009). Urinary exosomes contain approximately 3% of total urine protein based on a previous report (Zhou et al., 2006b) and thus provide a promising source for biomarker discovery because of the ability to detect proteins with relatively low abundance and the reduced complexity of the urinary proteome (Pisitkun et al., 2004; Wang et al., 2012). Several urine exosome-associated proteins with potential diagnostic value have been identified for other diseases, such as exosomal Fetuin-A protein in kidney injury (Zhou et al., 2006a), resistin, GTPase NRas, or galectin-3-binding protein in bladder cancer (Smalley et al., 2008), and PSA and PAMA in prostate cancer (Mitchell et al., 2009). However, the value and abundance of biological information of proteins contained in urinary exosomes deserve further investigation.

**FIGURE 1:**
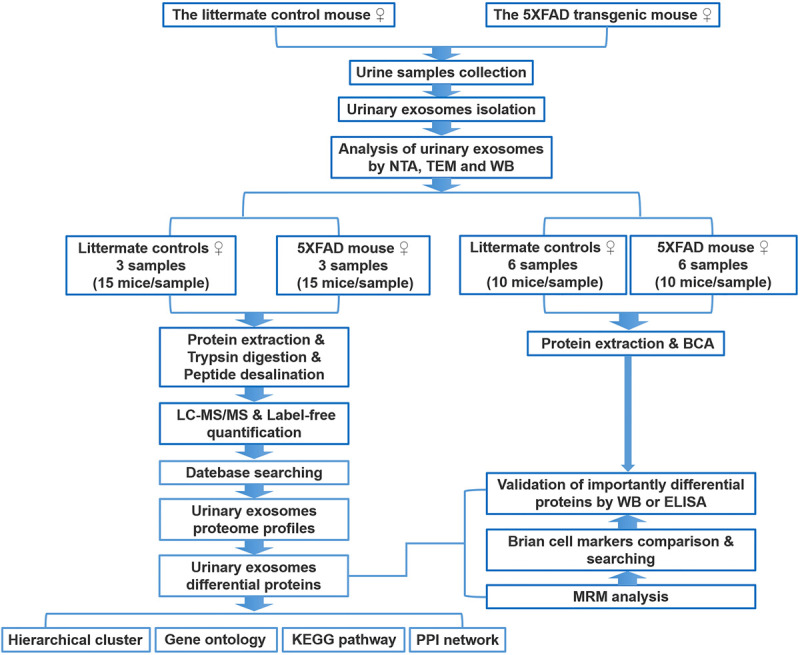
Workflow of isolation, characterization, and proteomics of urinary exosomes in 5XFAD transgenic mouse model of AD and littermate control. The proteins were analyzed using liquid chromatography coupled with tandem mass spectrometry (LC-MS/MS), MRM, WB, and ELISA. Differential proteins were analyzed by bioinformatics analysis. See text for details.

Exosomal proteomics has emerged as a powerful technique to understand the molecular constituent of exosomes and has the potential to accelerate biomarker discovery (Li et al., 2011). Animal models provide the necessary tools to overcome typical confounding variables, such as genetic heterogeneity, gender differences, and environmental factors, including diet and lifestyle, in clinical cases. 5XFAD mice rapidly recapitulate major features of AD amyloid pathology and exhibit intraneuronal Aβ_42_ accumulation in the brain at 1.5 months of age (before plaques form) (Oakley et al., 2006; Dinkins et al., 2014). Herein, we selected 5XFAD mice as an AD model to examine possible differentially expressed proteins in urinary exosomes. Specifically, urinary exosomes were collected and isolated from specific pathogen-free, 4-week-old, female 5XFAD mice and littermate controls to explore potentially significant information before the development of intraneuronal Aβ_42_ accumulation in the brain of 5XFAD mice (Figure 1). We performed, for the first time, urinary exosomal proteomic analysis combined with MRM, WB, and ELISA to explore early biomarkers of AD and provide an opportunity for prevention.

## Materials and Methods

### Animal Ethics Statement

All experimental procedures for the laboratory animals in this study were performed and approved according to the strict guidelines of Chinese Regulations of Laboratory Animals. All procedures in this study involving animals were reviewed and approved by the Institutional Animal Care and Use Committee of the Institute of Laboratory Animal Science, Peking Union Medical College (Approved ID:QC19005).

### Animals

Mice expressing 5 human mutations (5XFAD mouse model of AD) in APP and PS1(B6SJL-Tg[APP^∗^K670N^∗^M671L^∗^I716V^∗^V717I, PSEN1^∗^M146^∗^L286V] 6799Vas/J) under neuron-specific elements of the Thy1 promoter were purchased from The Jackson Laboratory and crossed to wild type SJL mice to generate offspring hemizygous for the APP and PS1 transgenes (Oakley et al., 2006). The positive mice tested by Polymerase Chain Reaction (PCR) (Transgene forward: AGG ACT GAC CAC TCG ACC AG; transgene reverse: CGG GGG TCT AGT TCT GCA T) were named as 5XFAD mice, littermate mice tested by PCR as negative are named as control group. All mice were maintained in an environmentally controlled room at 22°C on 12-h light/dark cycle and provided with standard diet and water *ad libitum*. These mice predominately generate Aβ_42_ that accumulates in plaques beginning at the age of 2 months. Urine samples from 4-week-old 5XFAD mice and littermate mice were collected in metabolic cages. During urine collection, all mice were given free access to water without food to avoid contamination.

### Purification of Exosomes

#### Urine Sample Preparation

Each urinary exosomes sample were isolated from 30 mL urine by Exosome Isolation Q3 kit (EIQ3-03001, Wayen Biotechnologies, Shanghai). The urine samples were taken from storage and kept it on ice. If starting with frozen sample, the samples were thawed completely in a 37°C water bath and then placed on ice. The urine samples were centrifuged at 3,000 × *g* for 15 min at 4°C. The supernatant was transferred to a new tube and placed on ice until ready to be performed for the isolation.

#### Exosomes Isolation

The Reagent A was balanced to room temperature before being used and the starting volume of urine was recommended to be 20 mL. The protocol below was shown with 20 mL urine. Twenty mL pre-treated urine was taken out, and added 7.5 mL Reagent A, the mixture was mixed well by inverting up and down untila homogenous mixture was obtained. Then 670 μL Reagent B was added to the mixture above and the tube was inverted up and down to obtain a homogenous mixture. The mixture was incubated at 4°C overnight (12–16 h). After incubation, it was centrifuged at 3,000 × *g* for 60 min at 4°C. One mL supernatant was taken out into a 1.5 mL tube firstly and thenthe residual supernatant was removed. The pellet was resuspended completely with the 1 mL supernatant above. The mixture was transferred to a new 1.5 mL tube. The re-suspension was centrifuged at 10,000 × *g* for 10 min at 4°C. The supernatant was removed without disturbing the precipitated pellet. The pellet with 50–200 μL 1 × PBS was resuspended, and it was mixed well by vortexing or pipetting up and down until a homogenous mixture was obtained. The sample was centrifuged at 10,000 × *g* again for 5 min at 4°C. The supernatant was transferred to a new tube. The supernatant contained exosomes. The exosomes can be used for downstream analysis immediately or aliquoted and stored at −80°C till next experiment.

### Label Free Analysis of Urinary Exosomal Proteins

#### Protein Extraction

Samples were lysed in moderate lysis buffer (2% SDS, 7M Urea) containing protease inhibitors (Thermo Fisher), followed by 1 min of sonication on ice using a ultrasonic processor (ultrasound on ice for 2 s, stop for 5 s), and rested on ice for 30 min. The lysate was centrifuged at 13,000 rpm and 4°C for 20 min, and the supernatants were collected. The protein concentration was determined using the BCA assay. Six times volume of 100% acetone was added and precipitated overnight at −20°C. The sample solutions were centrifuged at the next day. The precipitates were collected and washed twice by 500 μL washing buffer (ethanol: acetone: acetic acid = 50: 50: 0.1). Finally, after centrifugation at 13,000 rpm and 4°C for 15 min, the precipitates were dissolved and the protein concentration was quantified with BCA assay for the second time.

#### Trypsin Digestion

First, 100 μg protein solution samples were taken from each sample, and the volume was determined to 100 μL with 25 mM ammonium bicarbonate. Then, 1 M DTT was added (terminal concentration 20 mM), and the reduction reaction was kept for 1 h at 57°C. Subsequently iodoacetamide was added (terminal concentration 90 mM) and incubated for 40 min at room temperature under dark conditions. The sample solution was centrifuged on a 10 kDa ultrafiltration tube at 12,000 rpm, and ammonium bicarbonat was added into the ultrafiltration tube to wash four times. The sample was digested with trypsin which was diluted with ammonium bicarbonat at 37°C overnight. Next day the peptides were collected after centrifugation, and dried by centrifugal concentration.

#### Peptide Desalination

The dried peptides were desalted by Ziptip C18 column and analyzed by mass spectrometry. Desalination method are as follows: the dried mixed peptides were dissolved in 0.1% formic acid (FA) solution, and 100% acetonitrile was used to activate the desalting column, then 0.1% FA solution was used to balance the desalting column. The redissolved sample was added into the desalting column so that the sample flowed slowly through the desalting column, the peptide was captured by the desalting column, and other non-hydrophobic small molecules such as salt flowed out and abandoned. Then 0.1% FA solution was added to clean the desalting column and remove the residual salts. After 0.1% FA, 80% acetonitrile solution was added and slowly flow through the desalting column, the peptide was eluted and the elution solution was transferred to a new tube. The elution solution was concentrated and dried by centrifugation to remove acetonitrile.

#### Liquid Chromatography Tandem Mass Spectrometry

The dried sample was dissolved in buffer (acetonitrile: water: formic acid = 2: 98: 0.1), and analyzed by mass spectrometry. The on-line Nano-RPLC liquid chromatography was performed by Easy-nLC 1200 system (Thermo Scientific). The trap column was home-made C18 (C18, 5 um, 100 um^∗^2 cm) and the analytical column was C18 reversed-phase column (C18, 1.9 μm, 75 μm × 200 mm). The peptides results were subjected to nano electrospray ionization source followed by tandem mass spectrometry in Orbitrap Fusion Lumos (Thermo Fisher Scientific). The mass spectrometer was operated in the data-dependent mode. For MS scans, the scan ranged from 350 to 1,600 m/z. Intact peptides were detected at a resolution of 60,000 and peptides were then selected for MS/MS at a resolution of 15,000. Collision energy:30% HCD.

### Nanoparticle Tracking Analysis (NTA)

Nanoparticle tracking analysis was performed using a NanoSight instrument (PARTICLE METRIX Malvern Panalytical, Ltd., Malvern, United Kingdom) with a 488 nm laser and automated syringe pump. The ZetaView 8.04.02 software was used to process the recorded movies. The script was adapted as follow: samples diluted 1:100 in PBS were loaded using an automated syringe pump. Speed setting was initially fixed to 1000 for sample loading and chamber filling and then decreased to 25 for videos recording. A delay of 15 s was set to stabilize the flow before acquisition. Video captions of 60 s were done in triplicate for each sample with a camera level setting at 14 and a detection threshold at 3. PBS used for EVs recovery was used for negative controls (UC experiments). As a control for ODG experiments, 200 μL of PBS were loaded at the bottom of the tube that was then processed exactly in the same conditions as the urinary exosmes-containing samples.

### Transmission Electron Microscopy (TEM)

The observation of EVs by TEM was performed as previously described (Conde Vancells et al., 2010). Briefly, the isolated EVs were resuspended in 30 μL of 2% paraformaldehyde (PFA) in PBS. 3 × 10 μL of sample were deposited on Formvar-carbon-coated copper grids. The adsorption was performed for 3 × 20 min in a wet environment and then the grids were transferred into a drop of 1% glutaraldehyde in PBS for 5 min at room. After several rinsing steps with ultrapure water, samples were contrasted for 10 or 15 min on ice with a mixture of 4% uranyl acetate and 2% methylcellulose (1:9, v/v). The excess of mixture was removed using Whatman filter paper. After drying, samples were observed under a JEOL JEM-2100 TEM at 200 kV. The acquisitions were made with Gatan Orius SC200D camera.

### Data Analysis of Urinary Exosomal Proteome

The MS/MS data were analyzed with MaxQuant software (version 1.5.8.3, Max-Planck Institute for Biochemistry, Germany) (Tyanova et al., 2016), and proteins were identified by comparing the peptide spectra against the Swissprot databases (taxonomy: *Mus musculus*) (Table 3). Trypsin was selected as the digestion enzyme, up to two missed cleavage sites were allowed, and carbamidomethylation of a cysteine was defined as a fixed modification. The precursor ion mass tolerance and the fragment ion mass tolerance were 0.05 Da. Peptide identification was accepted at a false discovery rate (FDR) of less than 1.0% at the protein level and if the sample produced at least 2 unique peptides. The spectra counts of each protein were normalized by the total spectra counts of identified proteins. Data analysis was contract service offered by Wayen Biotechnologies (Shanghai), Inc. (Shanghai, China).

### Multiple Reaction Monitoring (MRM) Experiments and Data Analysis

Data derived from a spectral library of the normal urine exosome proteome generated by Label free LC-MS/MS using HCD collision were imported into Skyline version 19.1 (Vidova and Spacil, 2017). Skyline was employed to manually select the most intense peptide transitions. Up to five transitions per peptide were traced on a QTRAP 6500 mass spectrometer (AB Sciex). Peptides with potential modification sites (cysteine and methionine) and those with missed cleavage sites were excluded. A total of 2–5 peptides from one protein were selected for quantification. Using pooling sample to get the best chromotophy and mass parameter. Then we used MRM model to detect the samples. All the MRM data were imported into Skyline, which was used for further visualization, transition detection, and abundance calculation. The peptide abundance in a sample was calculated as the mean summed peak area of all selected transitions.

### Western Blotting

Extracted proteins were separated by SDS-PAGE on 10–15% gels, and then transferred onto a nitrocellulose membrane. Non-specific binding sites were blocked by 5% dried fat-free milk in Tris-buffered saline (TBS-T: 10 mmol/L Tris, 0.15 mol/L NaCl, 0.05% Tween-20, pH of the solution adjusted to 7.5). Primary antibodies (the antibodies used in this research were listed in Table 1 for exosome biomarkers and for targets proteins) were added and incubated at 4°C overnight as described previously (Song et al., 2017, 2018). Membranes were washed with TBST, and then incubated with the secondary antibody, either goat anti-mouse IgG or anti-rabbit IgG horseradish peroxidaseconjugated antibody (1:5000). Bands of immunoreactive proteins were visualized on an image system (Versadoc; Bio-Rad) after membrane incubation with enhanced chemifluorescence (ECF) reagent for 5 min as described previously (Vidova and Spacil, 2017).

**TABLE 1:** Antibodies used for testing the urinary exosome biomarker proteins.

**Protein**	**Antibody**
ALIX	Mouse monoclonal anti-ALIX antibody [3A9], abcam, ab117600
CD10	Rabbit monoclonal [EPR22867-118] to, abcam, ab256494
CD63	Rabbit monoclonal anti-CD63 antibody [EPR21151], abcam, ab217345
Flotillin 1	Rabbit monoclonal anti-Flotillin 1 antibody [EPR6041], abcam, ab133497
TSG101	Mouse monoclonal anti-TSG101 antibody [4A10], abcam, ab83
Albumin	Rabbit monoclonal anti-Albumin antibody [EPR20195], abcam, ab207327

### Enzyme Linked Immunosorbent Assay

The amount of protein in exosomes resuspended in PBS was measured using a BCA assay kit according to the manufacturer’s instructions using bovine serum albumin as standard protein. Similar protein amounts of urinary exosomes dissolved in a lysis buffer containing 300 mM NaCl, 50 mM Tris-Cl pH 7.4, 1 mM EDTA, and 0.5% Triton X-100 were analyzed following the manufacturer’s protocol. Exosome cargo proteins were quantified by ELISA kits for mouse Annexin 2 (NOVUS, NBP2-68223) and mouse Clusterin (R&D Systems^®^ -DuoSet ELISA, DY2747, 15 plates) according to the manufacturer’s instructions.

### Statistical Analysis

All assays were performed on three separate occasions. Data were expressed as means ± SD. We have checked the distribution of all datasets and all were parametric. All comparisons for parametric data were made using Student’s *t*-test or one-way ANOVA followed by *post hoc* Turkey’s test using the SPSS software (version 13.0: SPSS, Inc., Chicago, IL, United States), GraphPad Prism 5 software (La Jolla, CA, United States) and Image J software (National Institutes of Health, United States). *P* < 0.05 was considered statistically significant.

## Results

### Isolation and Characterization of Urinary Exosomes

**FIGURE 2:**
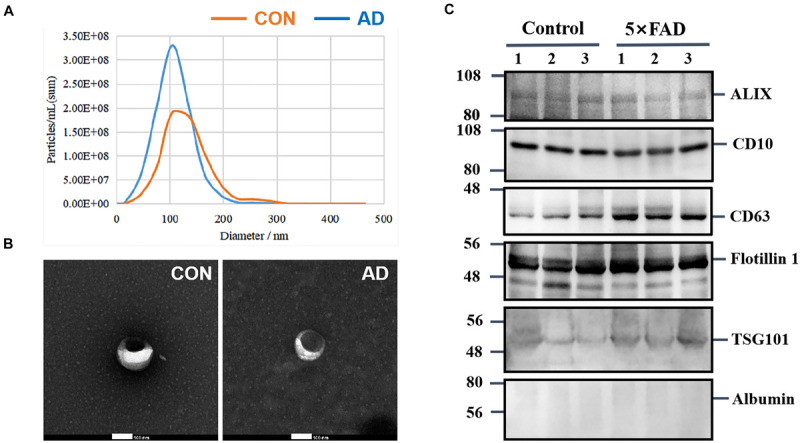
Characterization of urinary exosomes. **(A)** NTA of exosomes purified urinary exosomes. **(B)** The observation of exosomes by transmission electron microscopy (TEM). Bar = 100 nm. **(C)** After protein quantification, exosomes were lysed to perform immunoblot with specific monoclonal antibodies against typical exosome proteins were used to validate the quality of exosmes. Identification of Alix, CD10, CD63, Flotillin 1, and TSG101 in urinary exosomes (20 ug total protein per load) by Western blot, Albumin is a negative control protein were used to validate the quality of our isolation technique.

Urinary exosomes were isolated from 30 ml of urine in each sample (six samples, *n* = 15 mice per sample). Membrane vesicles less than 220 nm were purified from pooled urine samples by filtration and differential centrifugation. Quantitative analysis of urinary exosomes from the control and 5XFAD groups were tested by NTA (Table 2), which illustrated that the mean diameters of exosomes were 120.8 ± 43.0 nm (*n* = 642) and 103.2 ± 34.1 (*n* = 882), respectively (Figure 2A). The mean diameter of urinary exosomes in the 5XFAD group was smaller than that in the control group, but the concentration in the 5XFAD group was higher than that in the control group.

**TABLE 2:** The nanoparticle tracking analysis (NTA) of urinary exosomes.

**Sample name**	**Original concentration (Particles/mL)**	**Dilution factor**	**Test concentration (Particles/mL)**	**Particle size (nm)**	**Number of traced particles**	**Urinary volume (mL)**
Control	1.4E + 11	2400	5.8E + 7	120.8 ± 43.0	642	14.5
5XFAD	6.5E + 11	9600	6.8E + 7	103.2 ± 34.1	882	19.7

Morphologically, exosomes appear as flattened spheres or have a rounded erythrocyte-like shape as observed by a transmission electron microscope (Figure 2B). Equal amounts of protein derived from the urinary exosomes of each sample were subjected to WB analysis using antibodies that have been reported to show typical enrichment in urinary exosomal markers (Conde Vancells et al., 2010), such as the transmembrane proteins, CD10, CD63 and Flotillin 1, as well as antibodies to cytoplasmic proteins, such as Alix and TSG101. Consistent with previous research, WB analysis of the samples showed enriched content of Flotillin 1 protein compared with other proteins (Table 1); therefore, this protein could be a potentially stable biomarker for urinary exosomes. Albumin, a protein that has been previously reported in urine samples, was used to assess the influence of urine on exosomes (Figure 2C). These experiments indicate that the exosomes isolated from urine are relatively homogeneous, are not contaminated with urine, and contain exosomal markers.

### Proteomic Analysis of Urinary Exosomes in the 5XFAD and Control Groups

**FIGURE 3:**
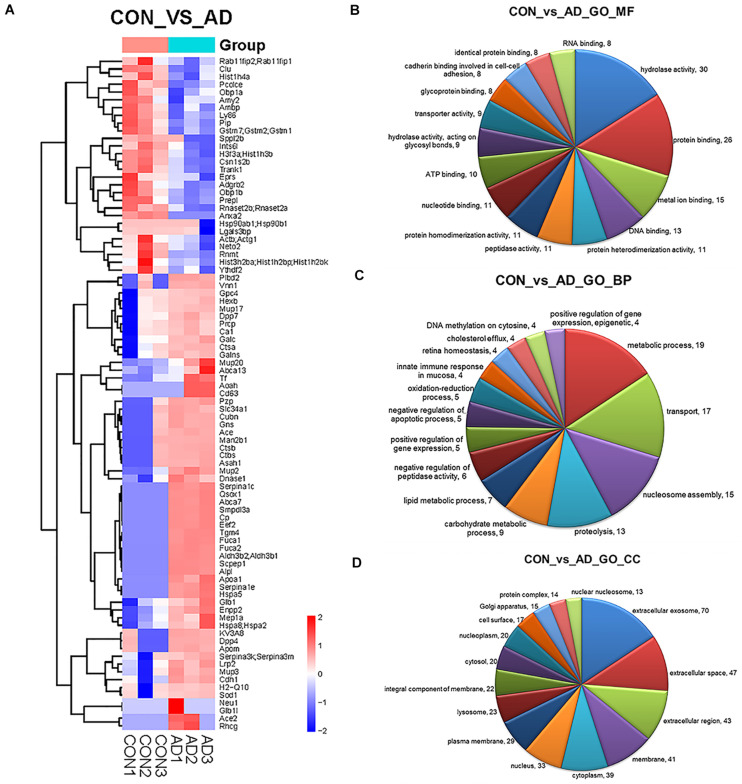
Comparison of the proteomics analysis by hierarchical cluster and gene ontology. **(A)** Heatmap illustrating the classification of control and 5XFAD mice (x axis) based on the deferentially expressed proteins (y axis) and unbiased clustering analysis in urinary exosomes. (**B–D)** Pie diagrams of Gene ontology (GO) analysis of the differentially expressed proteins was performed to study the specific MFs, BP, and CCs (the top 15 counts are shown) Comparison between control and 5XFAD mice samples in term of number of genes identified for different GO terms. Amounts of the top 15 terms with *P*-value < 0.05.

The proteins in urinary exosomes were measured by a BCA assay, and then samples containing 100 μg of exosomal protein were resolved by SDS-PAGE. The gel lanes were sliced, subjected to in-gel trypsinization and analyzed by LC-MS/MS. Raw data analysis by MaxQuant software (Table 3) permitted the identification of 316 proteins (Table 4) from six groups of samples contained in the *Swissprot* database at a false discovery rate < 1%. In total, 88 urinary exosomal proteins were significantly different between the 5XFAD and control groups based on the qualitative and quantitative analyses (Table 5 and Figure 3A). Compared with the control group, 53 proteins were up-regulated (ratio ≥ 1.2, *P*-value < 0.05) and 27 proteins were down-regulated (Ratio ≤ 0.83, *P*-value < 0.05) in the 5XFAD group according to precursor intensity quantitative analysis. The results were plotted and characterized as a heatmap showing differential hierarchical protein clusters with similar expression profiles (Figure 3A). Eighteen proteins were unique to the 5XFAD groups. In contrast, only one unique protein was found in the control group (Table 5). Among these differentially expressed proteins, 11 proteins were also cell markers in the brain and were selected for further study (Table 6). Specifically, at least 30 proteins had been reported to be associated with the pathological mechanisms or development of AD, while six were identified as direct AD biomarkers (Table 7) and had great potential to be considered as candidate urinary exosomal biomarkers of AD.

**TABLE 3:** The parameter values of the screening analysis of MS.

**Items**	**Parameter values**
Analysis software	MaxQuant
Database	Swissprot
Taxonomy	*Mus musculus*
Enzyme	Trypsin
Fixed modifications	Carbamidomethyl (C)
Variable modifications	Oxidation (M),Acetyl (Protein N-term)
Max Missed Cleavages	2
Peptide Charge State	2+,3+,4+
PSM FDR	0.01
Protein FDR	0.05
Site FDR	0.01
Min. peptide Length	7
MS/MS tol. (FTMS)	20 ppm
Decoy mode	Revert

**TABLE 4:** The screened number of urinary proteins in each samples.

**Items**	**Num.**
Total number of proteins (non-redundant)	316
Control-1	161
Control-2	153
Control-3	202
5XFAD-1	234
5XFAD-2	229
5XFAD-3	245
Total number of peptides (include redundant)	1364
Razor + unique peptides (number)	1253
Total number of protein spectrums (include redundant)	7457

**TABLE 5:** Details of differential urinary exosomal proteins between control and 5XFAD groups.

**Protein names**	**Majority protein IDs**	**Gene symbol**	**Unique peptides**	**Control (count)**	**5XFAD (count)**	**5XFAD vs. Control (Ratio)**	**5XFAD vs. Control (*P*-value)**	**References**
Low-density lipoprotein receptor-related protein 2	A2ARV4	Lrp2	79	3	3	2.60	0.05	
Serotransferrin	Q921I1	Tf	23	3	3	1.41	0.05	Haldar et al., 2013; Hare et al., 2015
Alpha-amylase 1	P00687	Amy1	22	3	3	0.61	0.05	Sramek et al., 1995
Pancreatic alpha-amylase	P00688	Amy2	22	3	3	0.61	0.05	
Meprin A subunit alpha	P28825	Mep1a	21	3	3	2.22	0.05	Bien et al., 2012
Alpha-2-macroglobulin	Q61838	Pzp	20	1	3	45.72	0.05	
Clusterin	Q06890	Clu	17	3	3	0.50	0.05	Liang et al., 2015; Vishnu et al., 2016; Miners et al., 2017
Ectonucleotide phosphodiesterase family member 2	Q9R1E6	Enpp2	15	3	3	8.24	0.05	Heywood et al., 2015
Angiotensin-converting enzyme	P09470	Ace	13	3	3	6.77	0.05	Rygiel, 2016
Beta-galactosidase	P23780	Glb1	13	3	3	4.04	0.05	
Beta-hexosaminidase subunit beta	P20060	Hexb	13	2	3	5.28	0.05	
Major urinary protein 20	Q5FW60	Mup20	13	3	3	2.03	0.05	
Cubilin	Q9JLB4	Cubn	12	1	3	12.28	0.05	
Lysosomal protective protein	P16675	Ctsa	11	3	3	2.84	0.05	Butler et al., 2011
Procollagen C-endopeptidase enhancer 1	Q61398	Pcolce	10	3	3	0.41	0.05	
Alpha-1-microglobulin	Q07456	Ambp	10	3	3	0.26	0.05	
*N*-acetylgalactosamine-6-sulfatase	Q571E4	Galns	9	2	3	7.54	0.05	
Serine protease inhibitor A3K; Serine protease inhibitor A3M	P07759; Q03734	Serpina3k; Serpina3m	9	2	3	9.59	0.05	Zhang et al., 2018
ATP-binding cassette sub-family A member 13	Q5SSE9	Abca13	8	3	3	3.08	0.05	
Dipeptidyl peptidase 2	Q9ET22	Dpp7	8	3	3	2.21	0.05	
Galactocerebrosidase	P54818	Galc	8	2	3	21.67	0.05	Filippov et al., 2012; Marshall and Bongarzone, 2016
Lysosomal alpha-mannosidase	O09159	Man2b1	8	1	3	3.22	0.05	
Major urinary protein 3	P04939	Mup3	8	3	3	1.73	0.05	
*N*-acetylglucosamine-6-sulfatase	Q8BFR4	Gns	8	1	3	10.01	0.05	
Actin, cytoplasmic 1, N-terminally processed; Actin, cytoplasmic 2, N-terminally processed	P60710; P63260	Actb; Actg1	7	3	3	0.56	0.05	
Cadherin-1	P09803	Cdh1	7	3	3	1.68	0.05	
Glypican-4	P51655	Gpc4	7	2	3	3.61	0.05	
Lysosomal Pro-X carboxypeptidase	Q7TMR0	Prcp	7	3	3	1.50	0.05	
Putative phospholipase B-like 2	Q3TCN2	Plbd2	7	1	3	16.73	0.05	
Odorant-binding protein 1b	A2AEP0	Obp1b	7	3	3	0.07	0.05	
Carbonic anhydrase 1	P13634	Ca1	6	3	3	4.09	0.05	
Cathepsin B	P10605	Ctsb	6	1	3	5.38	0.05	Hook et al., 2014; Sun et al., 2015
Deoxyribonuclease-1	P49183	Dnase1	6	3	3	2.90	0.05	
Dipeptidyl peptidase 4	P28843	Dpp4	6	1	3	4.47	0.05	
Histone H4	P62806	Hist1h4a	6	3	3	0.48	0.05	
Prolactin-inducible protein homolog	P02816	Pip	6	3	3	0.42	0.05	
Major urinary protein 2	P11589	Mup2	5	3	3	2.60	0.05	
Pantetheinase	Q9Z0K8	Vnn1	5	2	3	4.74	0.05	Zhang et al., 2018
Ribonuclease T2B; Ribonuclease T2A	C0HKG6; C0HKG5	Rnaset2b; Rnaset2a	5	3	3	0.68	0.05	
Acid ceramidase	Q9WV54	Asah1	4	1	3	4.45	0.05	Huang et al., 2004
Acid sphingomyelinase-like phosphodiesterase 3a	P70158	Smpdl3a	4	0	3	100.00	0.04	
Alpha-1-antitrypsin 1–5	Q00898	Serpina1e	4	2	3	100.00	0.04	Lieberman et al., 1995
Angiotensin-converting enzyme 2	Q8R0I0	Ace2	4	0	3	100.00	0.12	
Ceruloplasmin	Q61147	Cp	4	0	3	100.00	0.04	Kessler et al., 2006; Siotto et al., 2016
Di-*N-*acetylchitobiase	Q8R242	Ctbs	4	1	3	5.31	0.05	
Heat shock cognate 71 kDa protein;Heat shock-related 70 kDa protein 2	P63017; P17156	Hspa8; Hspa2	4	3	3	6.15	0.05	Kessler et al., 2006; Siotto et al., 2016
Plasma alpha-L-fucosidase	Q99KR8	Fuca2	4	0	3	100.00	0.04	
Sulfhydryl oxidase 1	Q8BND5	Qsox1	4	0	3	100.00	0.04	
Odorant-binding protein 1a	Q9D3H2	Obp1a	4	3	3	0.48	0.05	
78 kDa glucose-regulated protein	P20029	Hspa5	3	2	3	100.00	0.04	
Apolipoprotein A-I	Q00623	Apoa1	3	0	3	100.00	0.04	
Galectin-3-binding protein	Q07797	Lgals3bp	3	3	3	0.52	0.05	
H-2 class I histocompatibility antigen,	P01898	H2-Q10	3	2	3	2.93	0.05	
Lymphocyte antigen 86	O88188	Ly86	3	3	3	0.46	0.05	
Major urinary protein 17	B5 × 0G2	Mup17	3	3	3	8.44	0.05	
Protein-glutamine gamma-glutamyltransferase 4	Q8BZH1	Tgm4	3	0	3	100.00	0.04	
Retinoid-inducible serine carboxypeptidase	Q920A5	Scpep1	3	1	3	100.00	0.04	
Acyloxyacyl hydrolase	O35298	Aoah	2	0	3	100.00	0.12	
Alkaline phosphatase	P09242	Alpl	2	1	3	100.00	0.04	
ATP-binding cassette sub-family A member 7	Q91V24	Abca7	2	1	3	100.00	0.04	
Ig kappa chain V-III region PC 3741	P01660	Ig kappa chain V-III region PC 3741	2	1	3	7.20	0.05	
Sialidase-1	O35657	Neu1	2	0	3	100.00	0.32	
Signal peptide peptidase-like 2B	Q3TD49	Sppl2b	2	3	3	0.15	0.05	
Sodium-dependent phosphate transport protein 2A	Q60825	Slc34a1	2	1	3	5.94	0.05	
Superoxide dismutase [Cu-Zn]	P08228	Sod1	2	2	3	2.92	0.05	
Aldehyde dehydrogenase	J3QMK6	1700055N04Rik	1	0	3	100.00	0.04	
Alpha-1-antitrypsin 1–3	Q00896	Serpina1c	1	1	3	100.00	0.04	
Alpha-S2-casein-like B	P02664	Csn1s2b	1	3	3	0.34	0.05	
Ammonium transporter Rh type C	Q9QXP0	Rhcg	1	0	3	100.00	0.12	
Annexin A2	P07356	Anxa2	1	3	0	0.00	0.04	
Apolipoprotein M	Q9Z1R3	Apom	1	1	3	5.40	0.05	
Beta-galactosidase-1-like protein	Q8VC60	Glb1l	1	0	3	100.00	0.32	
Glutamate–tRNA ligase	Q8CGC7	Eprs	1	3	3	0.61	0.05	
Brain-specific angiogenesis inhibitor 2	Q8CGM1	Adgrb2	1	3	3	0.40	0.05	
CD63 antigen	P41731	Cd63	1	0	3	100.00	0.12	
Elongation factor 2	P58252	Eef2	1	0	3	100.00	0.04	
Glutathione *S*-transferase Mu 7	Q80W21	Gstm7	1	3	3	0.38	0.05	
Heat shock protein HSP 90-beta; Endoplasmin	P11499;P08113	Hsp90ab1;Hsp90b1	1	0	3	NA	NA	
Histone H2B type 3-A	Q9D2U9	Hist3h2ba	1	3	3	0.50	0.05	
Histone H3.3	P84244	H3f3a	1	3	3	0.29	0.05	
mRNA cap guanine-N7 methyltransferase	Q9D0L8	Rnmt	1	3	3	0.30	0.05	
Neuropilin and tolloid-like protein 2	Q8BNJ6	Neto2	1	3	3	0.30	0.05	
Prolyl endopeptidase-like	Q8C167	Prepl	1	3	3	0.11	0.05	
Protein DDX26B	Q8BND4	Ints6l	1	3	3	0.38	0.05	
Rab11 family-interacting protein 2;Rab11 family-interacting protein 1	G3XA57; Q9D620	Rab11fip2;Rab11fip1	1	3	3	0.65	0.05	
Tissue alpha-L-fucosidase	Q99LJ1	Fuca1	1	0	3	100.00	0.04	
TPR and ankyrin repeat-containing protein 1	Q8BV79	Trank1	1	3	3	0.31	0.05	
YTH domain-containing family protein 2	Q91YT7	Ythdf2	1	3	3	0.33	0.05	

*The ordering according to the number of unique peptides (descending order).*

**TABLE 6:** Differential proteins identified in urinary exosomes that are associated with cell makers of brain.

**Protein names**	**Majority protein IDs**	**Gene symbol**	**Unique peptides**	**Control (count)**	**5XFAD (count)**	**5XFAD vs. Control (Ratio)**	**5XFAD vs. Control (*P*-value)**
Ceruloplasmin	Q61147	Cp	4	0	3	100.00	0.04
Plasma alpha-L-fucosidase	Q99KR8	Fuca2	4	0	3	100.00	0.04
Acyloxyacyl hydrolase	O35298	Aoah	2	0	3	100.00	0.12
CD63 antigen	P41731	Cd63	1	0	3	100.00	0.12
Clusterin	Q06890	Clu	17	3	3	0.50	0.05
Ectonucleotide phosphodiesterase family member 2	Q9R1E6	Enpp2	15	3	3	8.24	0.05
Beta-hexosaminidase subunit beta	P20060	Hexb	13	2	3	5.28	0.05
Procollagen C-endopeptidase enhancer 1	Q61398	Pcolce	10	3	3	0.41	0.05
Lymphocyte antigen 86	O88188	Ly86	3	3	3	0.46	0.05
Prolyl endopeptidase-like	Q8C167	Prepl	1	3	3	0.11	0.05
Cathepsin B	P10605	Ctsb	6	1	3	5.38	0.05

**TABLE 7:** Differential proteins identified in urinary exosomes have been reported as direct AD biomarkers.

**Protein names**	**Majority protein IDs**	**Gene Symbol**	**Unique peptides**	**Control (count)**	**5XFAD (count)**	**5XFAD vs. Control (Ratio)**	**5XFAD vs. Control (*P*-value)**
Serotransferrin	Q921I1	Tf	23	3	3	1.41	0.05
Ectonucleotide phosphodiesterase family member 2	Q9R1E6	Enpp2	15	3	3	8.24	0.05
Galactocerebrosidase	P54818	Galc	8	2	3	21.67	0.05
Cathepsin B	P10605	Ctsb	6	1	3	5.38	0.05
Alpha-1-antitrypsin 1–5	Q00898	Serpina1e	4	2	3	100.00	0.04
Ceruloplasmin	Q61147	Cp	4	0	3	100.00	0.04

### Comparison of the Proteomics Analysis by Hierarchical Clusters and Gene Ontology, KEGG Pathway, and Protein–Protein Interaction (PPI) Network Analysis

**FIGURE 4:**
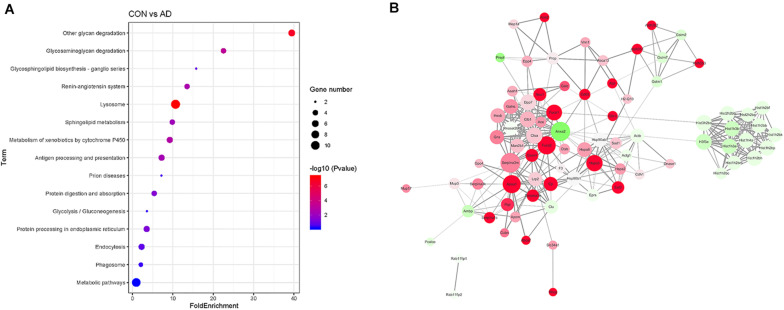
Comparison of the proteomics analysis by KEGG pathway and PPI network analysis. **(A)** Kyoto Encyclopedia of Genes and Genomes (KEGG) pathway analysis (the top 15 proteins with the greatest fold enrichment are shown). Dot plots of enriched KEGG pathways in differential proteins. The x axis shows the fold enrichment of each KEGG pathway, whereas the color denotes the *P*-value and the area of the dots represents the number of genes assigned to each KEGG pathway. The y axis displays KEGG pathway term. **(B)** PPI analysis showed the differential proteins are involved in different network. Red indicated upregulated genes, and green indicated downregulated genes in the present study.

Gene ontology (GO) analysis of the differentially expressed proteins was performed to study the specific molecular functions (MF), BP, and CCs (the top 15 counts are shown) (Figures 3B–D). Functional enrichment analyses based on GO terms revealed that differentially expressed urinary proteins mainly included extracellular exosomes or proteins associated with secretion of exosomes that participate in metabolic processes, transport, and gene expression. Kyoto Encyclopedia of Genes and Genomes (KEGG) pathway analysis (the top 15 proteins with the greatest fold enrichment are shown) (Figure 4A) (Table 8) showed that the enriched pathways within the cluster revealed that the differentially expressed proteins were mainly involved in (1) catabolic pathways, such as glycan degradation, protein digestion and absorption, and sphingolipid metabolism; (2) the immune system, such as antigen processing and presentation; and (3) cellular metabolism, such as lysosomes, endocytosis, and phagosomes. Interestingly, two genes, *HSPA5* and *SOD1*, involved in prion disease, which is also a neurodegenerative disease with pathogenesis similar to AD, were also enriched (Silva et al., 2014). Additionally, endoplasmic reticulum (ER) chaperone immunoglobulin binding protein (BiP/GRP78) which is encoded by *HSPA5* binds to and facilitates correct folding of the nascent cellular amyloid precursor protein (APP) (Yang et al., 1998). BiP/GRP78 also plays an essential role in protection from neuronal apoptosis (Wang et al., 2010). In contrast to the KEGG pathway analysis, GO term enrichment analyses revealed that GO terms associated with “metabolic process” and “immune response” were enriched. The above data demonstrated that significant changes involved in metabolic dysfunction and immune system disorders occurred even in the very early stage in 5XFAD mice. Determination of the PPI network offered a conceptual framework to better understand the functional organization of the differentially expressed urinary exosomal proteins with potential interactions (Figure 4B). Red indicated upregulated genes, and green indicated downregulated genes in the present study.

**TABLE 8:** Detail information of Kyoto Encyclopedia of Genes and Genomes (KEGG).

**Term**	**Count**	**Gene symbol**	***P-*value**	**Fold enrichment**
mmu00511:Other glycan degradation	6	HEXB, NEU1, FUCA2, MAN2B1, FUCA1, GLB1	0.00	39.44
mmu00531:Glycosaminoglycan degradation	4	GNS, HEXB, GALNS, GLB1	0.00	22.54
mmu00604:Glycosphingolipid biosynthesis – ganglio series	2	HEXB, GLB1	0.12	15.78
mmu04614:Renin–angiotensin system	4	ACE, ACE2, PRCP, CTSA	0.00	13.52
mmu04142:Lysosome	11	GNS, HEXB, GALNS, GALC, NEU1, CTSA, CTSB, CD63, MAN2B1, ASAH1, GLB1	0.00	10.67
mmu00600:Sphingolipid metabolism	4	GALC, NEU1, ASAH1, GLB1	0.01	9.86
mmu00980:Metabolism of xenobiotics by cytochrome P450	5	GSTM1, GSTM2, ALDH3B2, ALDH3B1, GSTM7	0.00	9.24
mmu04612:Antigen processing and presentation	5	HSP90AB1, H2-Q10, HSPA2, CTSB, HSPA8	0.00	7.21
mmu05020:Prion diseases	2	HSPA5, SOD1	0.24	7.17
mmu04974:Protein digestion and absorption	4	ACE2, MEP1A, PRCP, DPP4	0.04	5.38
mmu00010:Glycolysis/Gluconeogenesis	2	ALDH3B2, ALDH3B1	0.43	3.59
mmu04141:Protein processing in endoplasmic reticulum	5	HSP90AB1, HSP90B1, HSPA2, HSPA5, HSPA8	0.05	3.52
mmu04144:Endocytosis	5	RAB11FIP2, H2-Q10, HSPA2, RAB11FIP1, HSPA8	0.17	2.27
mmu04145:Phagosome	3	ACTG1, ACTB, H2-Q10	0.42	2.08
mmu01100:Metabolic pathways	10	GNS, ALPL, HEXB, GALNS, GALC, EPRS, ALDH3B2, ALDH3B1, ASAH1, GLB1	0.75	0.93

### Identification of Differentially Expressed Proteins by Multiple Reaction Monitoring (MRM) and WB

**FIGURE 5:**
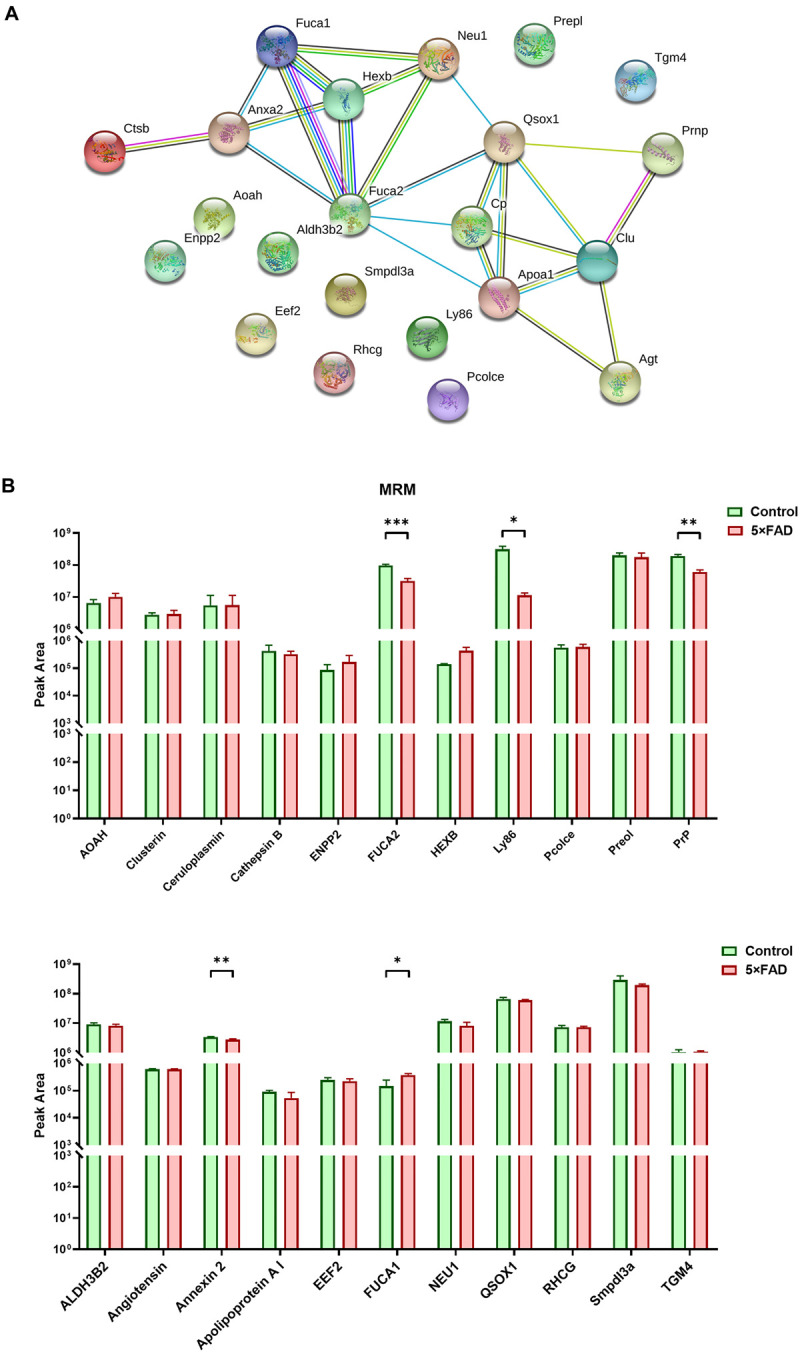
PPI analysis and multiple reaction monitoring (MRM) assays for the target proteins from control and 5XFAD mice. **(A)** PPI analysis showed the relationship of the target differential proteins. More lines between two proteins represented more possible interaction. **(B)** The relative peak area of 22 target proteins were measured by MRM between control and 5XFAD mice. All data are presented as mean ± SD in triplicate experiments; Student’s *t*-test; *P < 0.05; **P < 0.01; ***P < 0.001.

In the total identified proteins, 44 proteins are cell markers associated with the brain. Eighty-eight urinary exosomal proteins were significantly different between the 5XFAD and control groups based on the qualitative and quantitative analyses. Among them, 11 proteins are cell markers associated with the brain (Table 6). Fifth proteins that unique expression in different groups were selected. According to the results of the proteomic analysis, a total of 22 proteins were selected to do the next verification (Supplementary Figure 1). To make the relationship of targeted proteins more clear and intuitive, we further added the protein–protein interaction (PPI) network analysis of the 22 targeted proteins (Figure 5A). Next, MRM assays were showed in Figure 5B. The sequence and mass values of the surrogate peptides and their heavy version are summarized in Table 9. The peak area of surrogate peptides from each protein was calculated and the relative quantity was compared between control and 5XFAD mice groups. FUCA2 (*P* = 0.00075), Ly86 (*P* = 0.021), PrP (*P* = 0.0045), Annexin 2 (*P* = 0.0063), and Fuca1 (*P* = 0.038) showed significant different between two groups.

**TABLE 9:** Peptides used for MRM.

**Protein**	**Peptides**	**Retention time (min)**	**Average (Control)**	**Average (5XFAD)**	**5XFAD vs. Control (Ratio)**
Apolipoprotein A I	VQPYLDEFQK	22.76	165353.3333	97940	0.59231
	VAPLGAELQESAR	20.39	139050	81493.33333	0.58607
Cathepsin B	NFYNVDISYLK	32.46	311566.6667	263100	0.84444
	EIMAEIYK	20.76	332233.3333	229833.3333	0.69178
Clustrin beta chain	EIQNAVQGVK	10.51	819266.6667	839266.6667	1.02441
	IDSLLESDR	17.32	2971666.667	2805000	0.94391
	ASGIIDTLFQDR	36.1	1982666.667	2340666.667	1.18056
	ELLQSFQSK	19.74	5373666.667	5546666.667	1.03219
ENPP2	AGTFFWSVSIPHER	33.5	107856.6667	231066.6667	2.14235
	DIEHLTGLDFYR	32.68	58330	104130	1.78519
FUCA1	DLVGELGAAVR	32.23	122173.3333	299100	2.44816
	DGLIVPIFQER	38.7	168830	452166.6667	2.67824
HEXB	LQPALWPFPR	37.85	122180	509800	4.17253
	ILEIISSLK	32.71	155103.3333	347033.3333	2.23743
Pcolce	GFLLWYSGR	37.2	468533.3333	529966.6667	1.13112
	TGGLDLPSPPSGTSLK	26.36	735966.6667	742033.3333	1.00824
	GPILPPESFVVLYR	41.4	475566.6667	470500	0.98935
TGM4	VILNRPLQPQDELK	14.15	1062300	1072833.333	1.00992
ALBU	LVNELTEFAK	26.29	21086666.67	20106666.67	0.95353
	YLYEIAR	19.46	23026666.67	20140000	0.87464
	GAC[CAM]LLPK	13.73	4218000	4202333.333	0.99629
	AEFVEVTK	14.31	27013333.33	26776666.67	0.99124
	LVTDLTK	12.89	21760000	19983333.33	0.91835

**FIGURE 6:**
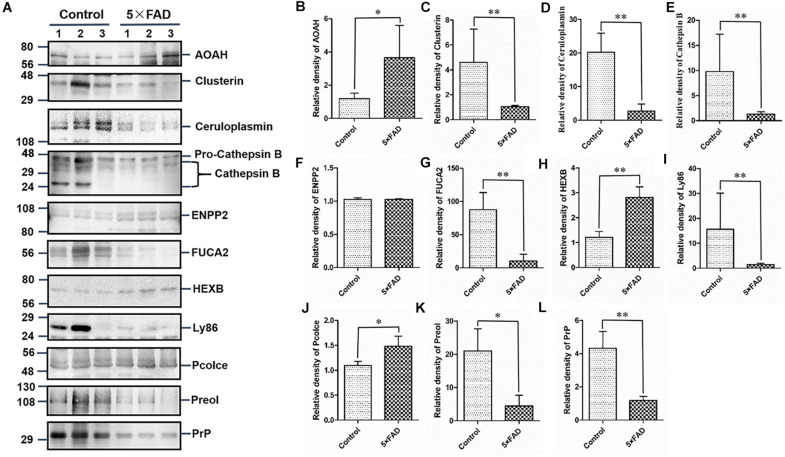
Western blots (WB) and quantitative analysis of 11 differential proteins in the urinary exosomes isolated from control and 5XFAD mice. **(A)** Same aliquot of urinary exosomes isolated from three control samples and three 5XFAD mice samples. AOAH, Clusterin, Ceruloplasmin, Cathepsin B, ENPP2, FUCA2, HEXB, Ly86, Pcolce, Preol, and PrP proteins were detected by WB in sequence. Representative blots of the tested proteins and the molecular weight of standard proteins were presented. **(B–L)** Quantitative analysis of each of the tested protein’s relative fold change, respectively. All data are presented as mean ± SD in triplicate experiments; Student’s *t*-test; *P < 0.05; **P < 0.01.

**FIGURE 7:**
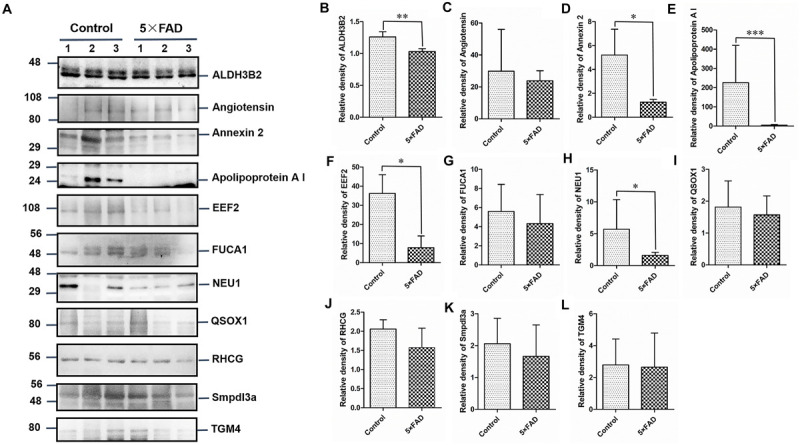
Western blots (WB) and quantitative analysis of the other 11 differential proteins in the urinary exosomes isolated from control and 5XFAD mice. **(A)** Same aliquot of urinary exosomes isolated from three control samples and three 5XFAD mice samples. ALDH3B2, Angiotensin, Annexin 2, Apolipoprotein A I, EEF2, FUCA1, NEU1, QSOX1, RHCG, Smpdl3a, and TGM4 proteins were detected by WB in sequence. Representative blots of the tested proteins and the molecular weight of standard proteins were presented. **(B–L)** Quantitative analysis of each of the tested protein’s relative fold change, respectively. All data are presented as mean ± SD in triplicate experiments; Student’s *t*-test; *P < 0.05; **P < 0.01.

All the 22 target proteins were analyzed by both MRM and WB (Figures 6A, 7A). The samples were displayed in the same loading order to confirm the above results by WB. The corresponding immunoblotting density of these proteins is shown in Figures 6B–L, 7B–L. Remarkably, according to the above three methods, HEXB, a lysosome and/or autophagy pathway protein, was significantly increased in the urinary exosomal proteins compared with the control group, consistent with the differential expression of HEXB in the brain of 5XFAD mice (Bai et al., 2020). Additionally, four proteins, AOAH, Clustrin, Ly86, and Preol, showed the same trend, with significant differences in the proteome and WB analyses. Compared with the control group, the AOAH level was significantly increased in the 5XFAD group; in contrast, the levels of Clustrin, Ly86, and Preol were obviously decreased. Based on the MRM and WB results, Apolipoprotein A1 and Cathepsin B were significantly reduced in 5XFAD mice compared with the control group, but these results are inconsistent with the proteomic analysis. However, although the quantitative WB analysis indicated no significant changes, proteome and MRM analyses demonstrated that the ENPP2 and Fuca1 levels in 5XFAD mice are higher than those in the control group. Although just five proteins were significant detected by MRM analyses, most of the other results showed the same trend to the results of WB. Independent of the other analysis, WB showed that in addition to the above mentioned proteins, seven proteins (ALDH3B2, Annexin-2, Ceruloplasmin, EEF2, FUCA2, PrP, and NEU1) were significantly decreased and one protein (Pcolce) was significantly increased in 5XFAD mice compared with the control group (Table 10). The sensitivity and accuracy vary in different methods, and these differentially expressed proteins need to be further explored based on their biological function.

**TABLE 10:** Detail information of the proteins that were validated by WB approach for primary screening.

**Protein Name**	**Gene**	**Proteomic data (5 × FAD/Control ratio)**	**MRM (5 × FAD/Control ratio)**	**WB (5 × FAD/Control ratio)**	**Molecular weight(kDa)**	**Antibody (Catalog; dilution rate)**	**Subcellular localization**	**Functions and pathways**
Acid sphingomyelinase-like phosphodiesterase 3a	Smpdl3a	100.00	0.67137	0.80720	50	Invitrogen (PA5-69826; 1:1000)	Secreted	Smpdl3a are lysosomal proteins that hydrolyze sphingomyelin to ceramide and phosphocholine.
ALDH3B2	Aldh3b2	100.00	0.90197	0.81747**	43	Invitrogen (PA5-37122; 1:1000)	-	Alcohol metabolism; ethanol degradation
Angiotensin	Agt	0.00	1.00128	0.79965	92	Abcam (ab108252; 1:1000)	Secreted and cell membrane	Carboxypeptidase which converts angiotensin I to angiotensin 1–9, a peptide of unknown function, and angiotensin II to angiotensin 1–7, a vasodilator. Also able to hydrolyze apelin-13 and dynorphin-13 with high efficiency. May be an important regulator of heart function.
Annexin-2/ANXA2	Anxa2	0.00	0.82834**	0.36211* (0.55642*)	38	Abcam (ab178677; 1:1000)	-	May act as a receptor for annexin II on marrow stromal cells to induce osteoclast formation.
AOAH	Aoah	100.00	1.56085	1.44910* (2.27331**)	62	Invitrogen (PA5-68081; 1:1000)	Secreted > extracellular space > extracellular matrix > basement membrane.	Calcium-regulated membrane-binding protein whose affinity for calcium is greatly enhanced by anionic phospholipids. It binds two calcium ions with high affinity. May be involved in heat-stress response.
Apolipoprotein A1	Apoa1	100.00	0.58919	0.02005***	25	Abcam (ab7614; 1:1000)	Secreted.	Participates in the reverse transport of cholesterol from tissues to the liver for excretion by promoting cholesterol efflux from tissues and by acting as a cofactor for the lecithin cholesterol acyltransferase (LCAT). As part of the SPAP complex, activates spermatozoa motility.
Cathepsin B	Ctsb	5.38	0.76811	0.13463**	37	Abcam (ab214428; 1:1000)	Lysosome Melanosome	Thiol protease which is believed to participate in intracellular degradation and turnover of proteins. Has also been implicated in tumor invasion and metastasis.
Ceruloplasmin	Cp	100	1.01426	0.13618**	122	Abcam (ab48614; 1:1000)	Secreted	Ceruloplasmin is a blue, copper-binding (6–7 atoms per molecule) glycoprotein. It has ferroxidase activity oxidizing Fe(2+) to Fe(3+) without releasing radical oxygen species. It is involved in iron transport across the cell membrane
Clustrin beta chain	Clu	0.50	1.04527	0.51304** (0.4702***)	52	Abcam (ab184099; 1:1000)	Secreted	Isoform 1 functions as extracellular chaperone that prevents aggregation of non-native proteins. Prevents stress-induced aggregation of blood plasma proteins.
EEF2	Eef2	100.00	0.90817	0.21584*	95	Abcam (ab75748; 1:1000)	Cytoplasm	Catalyzes the GTP-dependent ribosomal translocation step during translation elongation. During this step, the ribosome changes from the pre-translocational (PRE) to the post-translocational (POST) state as the newly formed A-site-bound peptidyl-tRNA and P-site-bound deacylated tRNA move to the P and E sites, respectively.
ENPP2/ATX	Enpp2	8.24	1.96377	0.99887	99	Invitrogen (PA5-85221; 1:1000)	Secreted	Hydrolyzes lysophospholipids to produce lysophosphatidic acid (LPA) in extracellular fluids. Major substrate is lysophosphatidylcholine. Also can act on sphingosylphosphphorylcholine producing sphingosine-1-phosphate, a modulator of cell motility.
Fuca1	Fuca1	100.00	2.5632*	0.77161	54	Abcam (ab181357; 1:1000)	Lysosome	Alpha-L-fucosidase is responsible for hydrolyzing the alpha-1,6-linked fucose joined to the reducing-end *N-*acetylglucosamine of the carbohydrate moieties of glycoproteins.
FUCA2	Fuca2	100.00	0.32781***	0.12117**	54	Invitrogen (PA5-70565; 1:1000)	Secreted	Alpha-L-fucosidase is responsible for hydrolyzing the alpha-1,6-linked fucose joined to the reducing-end *N-*acetylglucosamine of the carbohydrate moieties of glycoproteins.
HEXB	Hexb	5.28	3.0294	2.32591**	63	Invitrogen (PA5-36146; 1:1000)	Lysosome	Responsible for the degradation of GM2 gangliosides, and a variety of other molecules containing terminal *N*-acetyl hexosamines, in the brain and other tissues.
MD1	Ly86	0.46	0.03629*	0.09264** (0.53287**)	27	Abcam (ab45424; 1:1000)	Secreted > extracellular space	May cooperate with CD180 and TLR4 to mediate the innate immune response to bacterial lipopolysaccharide (LPS) and cytokine production. Important for efficient CD180 cell surface expression.
PCOC1	Pcolce	0.41	1.0429	1.35342*	49	Invitrogen (PA5-47276; 1:1000)	Extracellular space, extracellular matrix, extracellular exosome	Binds to the C-terminal propeptide of type I procollagen and enhances procollagen C-proteinase activity
PPCEL/PREOL	Preol	0.11	0.87129	0.21226*	72	Abcam (ab203111; 1:2000)	Cytoplasm > cytosol	Probable serine peptidase whose precise substrate specificity remains unclear. Does not cleave peptides after a arginine or lysine residue.
PrP	-		0.31791**	0.27516**	30	Santa ceuz (sc-69896; 1:200)	Cell membrane	Characteristic of prion diseases, cellular PrP (PrP^c^) is converted to the disease form, PrP^Sc^, through alterations in the protein folding conformations.
QSOX1	Qsox1	100.00	0.91527	0.86572	83	Invitrogen (PA5-38009; 1:1000)	Golgi apparatus membrane and Secreted > extracellular space.	Catalyzes the oxidation of sulfhydryl groups in peptide and protein thiols to disulfides with the reduction of oxygen to hydrogen peroxide. May contribute to disulfide bond formation in a variety of secreted proteins. In fibroblasts, it may have tumor-suppressing capabilities being involved in growth regulation.
RHCG	Rhcg	100.00	0.99824	0.76588	53	Invitrogen (PA5-95693; 1:1000)	Apical cell membrane.	Functions as an electroneutral and bidirectional ammonium transporter. May regulate transepithelial ammonia secretion.
Sialidase-1	NEU1	100.00	0.70012	0.28070* (0.23653)	45	Invitrogen (PA5-42552; 1:1000)	Lysosome membrane.	Catalyzes the removal of sialic acid (*N*-acetylneuramic acid) moities from glycoproteins and glycolipids. To be active, it is strictly dependent on its presence in the multienzyme complex. Appears to have a preference for alpha 2–3 and alpha 2–6 sialyl linkage.
TGM4	Tgm4	100.00	1.00992	0.95013	75	Invitrogen (PA5-42106; 1:1000)	Cytoplasm, Golgi apparatus, integral component of membrane, extracellular matrix, extracellular exosome.	Associated with the mammalian reproductive process.

*All data are presented as mean ± SD in triplicate experiments; Student’s t test; ^∗^P < 0.05; ^∗∗^P < 0.01; ^∗∗∗^P < 0.001.*

### Further Validation of Urinary Exosomal Proteins Showed Significant Differences Between 5XFAD and Control Mice

After comprehensive evaluation of the proteome data, bioinformatics analysis, MRM, and WB, an independent set of 12 samples (*n* = 10 mice per sample) from the two groups was collected for further validation of the proteins as potential biomarkers in urinary exosomes.

**FIGURE 8:**
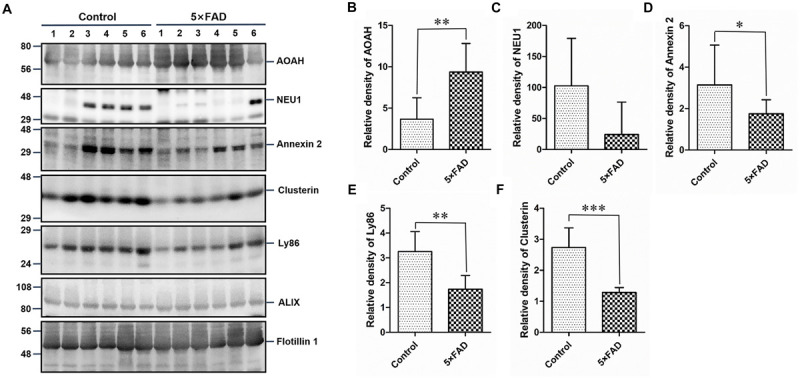
Western blots (WB) and quantitative analysis of an independent set of the urinary exosomes isolated from control and 5XFAD mice. **(A)** Same aliquot of urinary exosomes isolated from six control samples and six 5XFAD mice samples. AOAH, NEU1, Annexin 2, Clusterin, and Ly86 proteins were further detected by WB in sequence. Alix and Flotillin 1 were tested as urinary exosomes biomarkers. Representative blots of the tested proteins and the molecular weight of standard proteins were presented. **(B–F)** Quantitative analysis of each of the tested protein’s relative fold change, respectively. All data are presented as mean ± SD in triplicate experiments; Student’s *t*-test; *P < 0.05; **P < 0.01; ***P < 0.001.

**FIGURE 9:**
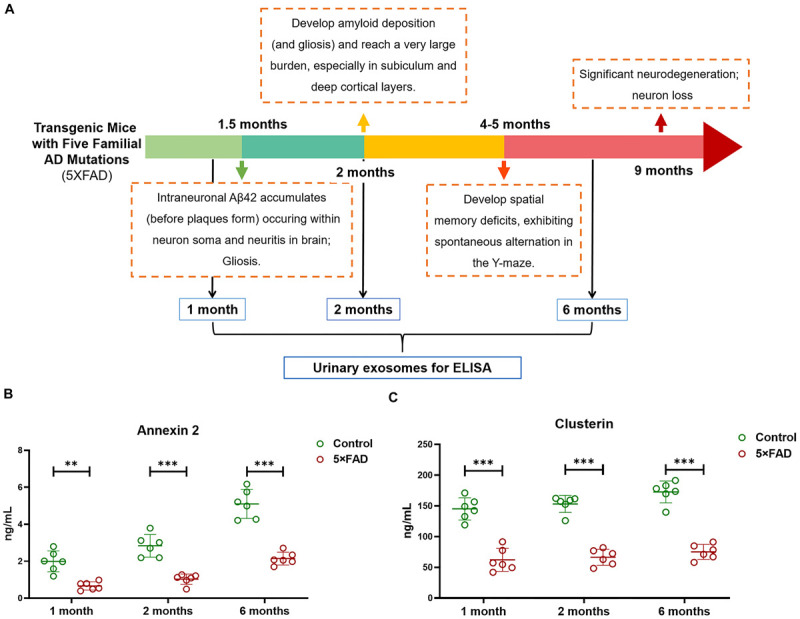
**(A)** The schematic diagram of the pathological feature of 5XFAD mice model during the AD progression. **(B,C)** Quantification of Annexin 2 and Clusterin in urinary exosome samples using ELISA from 1, 2, and 6-month-old 5XFAD mice and their littermate control. Each point represents value for one sample, and horizontal line in point clusters depicts mean level for that group. All data are presented as mean ± SD in triplicate experiments; Student’s *t*-test; **P < 0.01; ***P < 0.001.

Again, based on the WB analysis with the same loading amount, AOAH, NEU1, Annexin 2, Clusterin, and Ly86 levels were further confirmed to be significantly different and consistent with the initial WB results. Alix and Flotillin1 were detected as urinary exosomal markers (Figure 8A). The AOAH level was 2.27-fold higher in 5XFAD mice than in control mice. In contrast, the NEU1, Annexin 2, Clusterin, and Ly86 levels, exhibited 77, 45, 50, and 90% reductions, respectively, compared with the control group (Figures 8B–F). The pathological features of 5XFAD mouse showed in Figure 9A, at 2 months, the brain from 5XFAD mouse develops amyloid deposition and reaches a very large burden, and after 4–5 months, the 5XFAD mouse develops spatial memory deficits. Herein, 1, 2, and 6 month-old mouse were selected to collect their urinary for monitoring the dynamic change of target proteins during AD progression compared with their littermate controls. Two commercially available ELISA kits were used to detect the concentration of mouse Annexin 2 and Clusterin.

Firstly, urinary exosomes were isolated from 30 ml of urine in each urinary sample. For 1, 2, and 6-month-old time points, the urinary exosomes proteins were compared between 5XFAD mice (*N* = 6 samples per time point) and control mice (*N* = 6 samples per time point), respectively. Consist with the analysis of WB, the result of ELISA from the urinary exosomes of 1-month-old mice demonstrated that the concentrations of Annexin 2 (*P* = 0.0013) and Clusterin (*P* = 1.5E-05) in the control group were significantly higher than that in 5XFAD mice group. Moreover, during the AD progression, a longitudinal study was analyzed. Both at 2 and 6 months, the concentrations of Annexin 2 (*P* = 0.00031 and *P* = 7.1E-05, respectively) and Clusterin (*P* = 5.7E-07 and *P* = 1.7E-06, respectively) from urinary exosomes in the control group were extremely significant higher than that in 5XFAD mice group (Figures 9B,C). During the course, the levels of Annexin 2 and Clusterin were still lower in 5XFAD mice than that in matched control further suggesting that they could be the non-invasive source of candidates for the prevention of AD.

Remarkably, Clusterin was significantly decreased in the 5XFAD mice, which contrasts the results of previous research (Bai et al., 2020). Clusterin levels were higher in AD patients than in controls, especially in regions with the greatest abundance of Aβ, facilitating the development of AD (Miners et al., 2017). Importantly, a recent report indicated that three proteins, Aβ, TREM2 and Clusterin, display an early increase in patients with severe Aβ pathology but no detectable cognitive defects (Nelson et al., 2012) and in patients with mild cognitive impairment, with continuous accumulation in AD. These proteins are enriched in 12 signaling pathways, including the cytoskeleton and extracellular matrix, membrane transport, and synaptic function (Bai et al., 2020). As a heterodimeric glycoprotein, Clusterin is more abundant in the CSF of some neurodegenerative disease patients than in healthy controls, and could thus serve as a potential biomarker to differentiate Parkinson’s disease from dementia with Lewy bodies (Vranová et al., 2016). All of the evidence demonstrates that the detectable significant difference of Clusterin in urinary exosomes may be an ideal early and non-invasive biomarker to reflect AD pathology. The contrasting levels of Clusterin in the urinary exosomes of 5XFAD mice and in the brains of AD patients may occur because of the increased expression of Clusterin around Aβ plaques, leading to decreased Clusterin in the peripheral blood, resulting in reduced levels in urinary exosomes.

## Discussion

Current AD detection methods mainly include the determination of Aβ, tau, or phosphorylated tau levels in the CSF with invasive diagnostic procedures (He et al., 2010) and imaging techniques such as functional magnetic resonance imaging or positron emission tomography scans (Hare et al., 2015) during the late stages of the disease. Therefore, early diagnosis of AD is urgently required even before the pre-clinical phase to enhance the effects of therapeutic intervention. One challenge is often poor diagnostic specificity or sensitivity, which can be overcome in some instances by combining biomarkers. Biochemical and proteomic characterization of highly purified urinary exosomes identified 88 differentially expressed proteins by comparing 5XFAD and control mice. Among these, 22 proteins were validated by WB, and 15 proteins showed significant changes. Then, five proteins were further examined in an independent set of 12 groups of samples. Many of these proteins have been previously associated with AD but never reported in the urinary exosomal proteins of an AD mouse model. Furthermore, a portion of the proteins further selected for WB detection showed no significant changes, which may have occurred because WB analysis could not detect the limited amounts and concentrations of these proteins in urine exosome samples. In future researches, clinical urinary exosomes samples of AD patients will provide more significant data to validate the differential proteins identified in present study.

AOAH and Ly86 are also brain cell markers, as confirmed by single-cell profiling (Rosenberg et al., 2018). Clusterin has been previously reported to be related to the development of AD as well as a biomarker of AD (Pandey et al., 2019). They displayed the same trend, with significant differences in the proteome and first WB analyses. Alteration of the family of Annexin proteins, including Annexin 2, was observed in pathological neuronal and glial reactions from autopsy cases representing by hypoxic-ischemic injury, seizure disorders, and AD in the human hippocampus (Eberhard et al., 1994). In AD, astrocytic Annexin 2 positive protein was localized to discrete plaque-like areas (Eberhard et al., 1994). Annexin 2 is also related to tissue plasminogen activator mediated microglial activation during injury of the brain (Siao and Tsirka, 2002). *N*-acetyl-alpha-neuraminidase 1 (NEU1), a lysosomal sialidase, also called sialidase 1, is encoded by the *NEU1* gene. Growing evidence suggests that mammalian sialidases play an important role in the development and function of the CNS owing to the nervous tissue is the organ with the highest expression level of sialic acids (Liao et al., 2020). Additionally, chronic stress affects hippocampus-depended spatial learning of rat in the Barnes maze via sialidase activity (Wielgat et al., 2011). Slight changes might be detected by urinary via blood circulation system and metabolism in pathological environments.

Among the differentially expressed proteins, Ectonucleotide pyrophosphatase/phosphodiesterase family member 2 (ENPP2) levels were higher in the CSF of AD patients than the CSF of healthy controls. ENPP2 can be used to specifically discriminate AD from Lewy body dementia, making it a candidate AD biomarker (Heywood et al., 2015). In our study, proteomic and MRM analyses confirmed 8.24- and 1.96-fold increases in ENPP2 in 5XFAD mice, respectively, compared with the control group, but WB analysis showed no significant difference; thus, further confirmation by other methods is required.

Ceruloplasmin (CERU) levels were higher in the control group than in the 5XFAD mice, and the opposite trend of increasing levels was found in the serum of AD patients (Park et al., 2014), but the results were consistent with the CERU levels in the CSF of AD patients (Park et al., 2014). The differences in test results of different body fluids may have occurred because these fluids undergo different metabolic pathways and stages; however, all data showed significant differences. The ratio of CERU concentrations measured by enzymatic methods to those measured by immunological methods (iCP), eCP/iCP, reflects the high specificity of this protein in AD patients as well as a decreased risk of having AD (Siotto et al., 2016). Moreover, CERU had less ferroxidase activity in AD patients than in healthy patients, which also contributes to the development of AD (Bush, 2013).

Cathepsin B levels were also higher in the control group than in the 5XFAD mice according to WB analysis, which is opposite to the proteomic data results. Cathepsin B has been previously reported to be upregulated in brain tissues from APP/PS1 transgenic mice, and changes in Cathepsin B showed a similar direction in healthy mice and the serum of AD patients relative to control patients, making Cathepsin B a potential biomarker of AD (Sun et al., 2015). In addition, Cathepsin B produces brain pyroglutamate A, which represents a potential therapeutic agent for AD (Hook et al., 2014).

Prion protein (PrP) is a cell surface protein widely expressed in a variety of tissues in the central and peripheral nervous system. PrP is best known for its crucial role as a molecular substrate in the pathogenesis of prion diseases such as Creutzfeldt-Jakob disease and familial fatal insomnia in humans. Previous research has reported that PrP is detectable in normal rat urinary exosomes (Conde Vancells et al., 2010). Interestingly, PrP was significantly decreased in 5XFAD mice compared with the control group, although this protein was undetectable in the proteomic data. Several studies have demonstrated that certain misfolded amyloid proteins composed of Aβ, tau, or α-synuclein can be transferred among neurons, suggesting the contribution of mechanisms similar to those by which infectious prions spread through the brain (Conde Vancells et al., 2010). PrP may be a putative receptor and bind to Aβ oligomers, and PrP-dependent Aβ toxicity partially participates in the progression of AD (José et al., 2018; Purro et al., 2018). This finding could open new avenues to investigate the clearance mechanism of Aβ during AD.

## Conclusion

These findings support that some urinary exosomal proteins in 5XFAD mouse models enable detection of differences, providing potential early biomarkers before Aβ accumulation for improved diagnosis and prognosis prediction of AD.

## Data Availability Statement

The datasets presented in this study can be found in online repositories. The names of the repository/repositories and accession number(s) can be found below: https://www.ebi.ac.uk/pride/archive, accession no: PXD021935.

## Ethics Statement

The animal study was reviewed and approved by The Institutional Animal Care and Use Committee of the Institute of Laboratory Animal Science, Peking Union Medical College (Approved ID: QC19005).

## Author Contributions

CQ and ZS designed the project and wrote the manuscript. ZS, YX, LZa, LZo, YZ, XL, YH, PY, YQ, and WZ performed majority of the experiments. All authors analyzed the data.

## Conflict of Interest

The authors declare that the research was conducted in the absence of any commercial or financial relationships that could be construed as a potential conflict of interest.

## Supplementary Material

Supplementary Figure 1 The schematic diagram of how to select the target proteins for further identification.

## Abbreviations

Aβ: amyloid-β
AD: Alzheimer’s disease
BP: biological processes
CCs: cellular components
CERU: ceruloplasmin
CSF: cerebrospinal fluid
ELISA: enzyme-linked immunosorbent assay
ENPP2: ectonucleotide pyrophosphatase/phosphodiesterase family member 2
ER: endoplasmic reticulum
GO: gene ontology
KEGG: Kyoto Encyclopedia of Genes and Genomes
MFs: molecular functions
MRM: multiple reaction monitoring
NTA: nanoparticle tracking analysis
PrP: prion protein
WB: western blotting.
