# Supplementary figures and images for: Comprehensive Proteomic Profiling of Urinary Exosomes and Identification of Potential Non-invasive Early Biomarkers of Alzheimer’s Disease in 5XFAD Mouse Model
Source: Front Genet. 2020 Nov 5;11:565479. doi: 10.3389/fgene.2020.565479 (PMC7674956; doi:10.3389/fgene.2020.565479)

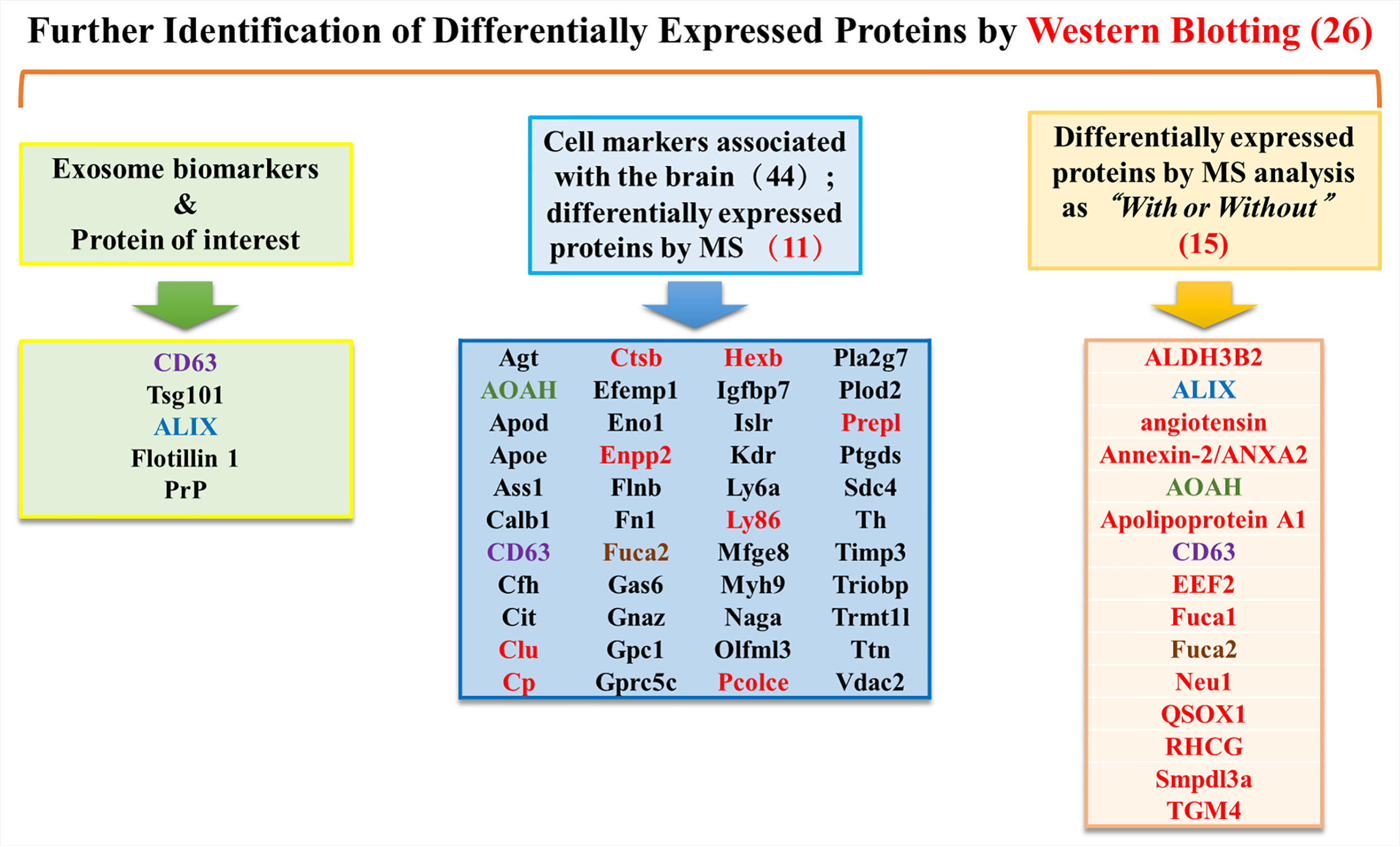

Supplement: Supplementary Figure 1 — The schematic diagram of how to select the target proteins for further identification. [file Image_1.tif]
